# Peripheral inflammation in behavioural variant frontotemporal dementia: associations with central degeneration and clinical measures

**DOI:** 10.1186/s12974-023-02746-5

**Published:** 2023-03-08

**Authors:** Min Chu, Lulu Wen, Deming Jiang, Li Liu, Haitian Nan, Ailing Yue, Yingtao Wang, Yihao Wang, Miao Qu, Ningqun Wang, Liyong Wu

**Affiliations:** Department of Neurology, Xuanwu Hospital, Capital Medical University, Beijing, 100053 China

**Keywords:** Inflammation, Behavioural variant fronto-temporal dementia, Neurodegeneration

## Abstract

**Background:**

Neuroinflammation plays a significant role in the progression of frontotemporal dementia (FTD). However, the association between peripheral inflammatory factors and brain neurodegeneration is poorly understood. We aimed to examine changes in peripheral inflammatory markers in patients with behavioural variant FTD (bvFTD) and explore the potential association between peripheral inflammation and brain structure, metabolism, and clinical parameters.

**Methods:**

Thirty-nine bvFTD patients and 40 healthy controls were enrolled and underwent assessment of plasma inflammatory factors, positron emission tomography/magnetic resonance imaging, and neuropsychological assessments. Group differences were tested using Student’s *t* test, Mann‒Whitney *U* test, or ANOVA. Partial correlation analysis and multivariable regression analysis were implemented using age and sex as covariates to explore the association between peripheral inflammatory markers, neuroimaging, and clinical measures. The false discovery rate was used to correct for the multiple correlation test.

**Results:**

Plasma levels of six factors, including interleukin (IL)-2, IL-12p70, IL-17A, tumour necrosis superfamily member 13B (TNFSF/BAFF), TNFSF12 (TWEAK), and TNFRSF8 (sCD30), were increased in the bvFTD group. Five factors were significantly associated with central degeneration, including IL-2, IL-12p70, IL-17A, sCD30/TNFRSF8, and tumour necrosis factor (TNF)-α; the association between inflammation and brain atrophy was mainly distributed in frontal–limbic–striatal brain regions, whereas the association with brain metabolism was mainly in the frontal–temporal–limbic–striatal regions. BAFF/TNFSF13B, IL-4, IL-6, IL-17A and TNF-α were found to correlate with clinical measures.

**Conclusion:**

Peripheral inflammation disturbance in patients with bvFTD participates in disease-specific pathophysiological mechanisms, which could be a promising target for diagnosis, treatment, and monitoring therapeutic efficacy.

**Supplementary Information:**

The online version contains supplementary material available at 10.1186/s12974-023-02746-5.

## Background

Behavioural variant frontotemporal dementia (bvFTD) is the most common clinical subtype of frontotemporal dementia (FTD) [1]. The underlying mechanism of bvFTD is the accumulation and deposition of abnormal pathological proteins, especially in the frontal–temporal brain regions, including tauopathy (TAU), TAR DNA-binding protein of 43 kDa (TDP-43), and fused-in-sarcoma (FUS) [2]. Chronic neuroinflammation also plays a significant role in FTD, which is a common secondary pathway after deposition of misfolded pathological proteins together with cellular debris.

Several studies that include animal models [3–5], human fluid biomarkers [6–8], or brain tissue immunohistochemistry [9–11] have provided evidence that an ongoing inflammatory process is involved in the progression of sporadic or genetic FTD, manifesting as microglial activation/dystrophy, astrogliosis, and increased secretion of inflammatory markers, including tumour necrosis factor (TNF) and interleukin (IL) cytokines [12, 13]. In patients with FTD, increased expression of both intrathecal and peripheral inflammatory markers was noted [14]. Fronto-temporal neurodegeneration, including atrophy or hypometabolism, has also been demonstrated in FTD; however, the direct association between peripheral inflammation and brain degeneration has not been well-investigated. Only one previous study discovered a significant role of peripheral inflammatory factors from the TNF family in modulating specific central disease-related structural abnormalities in patients with bvFTD [15]. In addition, inflammatory mediators modulate brain development and synapse plasticity [16, 17]; synaptic dysfunction can be sensitively reflected by brain metabolic alterations. To the best of our knowledge, the association between peripheral inflammatory markers and brain metabolism abnormalities has not been explored.

In this study, patients with bvFTD and healthy controls underwent assessment of peripheral inflammatory markers, hybrid positron emission tomography/magnetic resonance imaging (PET/MRI), and neuropsychological examinations. The aims of the study were as follows: (1) to explore the potential association between peripheral inflammation and central brain structure and metabolism and (2) to determine the relationship between peripheral inflammation and clinical parameters. We hypothesized that abnormal peripheral inflammatory markers (increased proinflammatory markers and decreased anti-inflammatory markers) would be associated with disease-specific central neurodegeneration and clinical alterations.

## Methods

### Ethics

This study was conducted following the Declaration of Helsinki. The clinical protocols were approved by the ethics committee and local institutional review board of Xuanwu Hospital, Capital Medical University, China. The study was conducted following relevant guidelines and regulations for the use of human subjects in research. Written informed consent was obtained from all participants or their guardians before the start of the study.

### Participants

Seventy-nine right-handed subjects were enrolled in this study, including 39 bvFTD patients, from July 1, 2014, to October 31, 2021, in the Department of Neurology of Xuanwu Hospital. All patients were diagnosed with probable bvFTD according to the consensus criteria published in 2011 [18]. Age- and sex-matched healthy controls who had no complaints of cognitive decline, depression, or anxiety and performed within the normal range on neuropsychological tests (Mini-Mental State Examination [MMSE] score ≥ 24 and Clinical Dementia Rating Scale [CDR] score = 0) were enrolled from communities.

Exclusion criteria for all participants were as follows: (1) any serious neuropsychiatric disorder that could affect cognitive function, such as substance abuse, alcoholism, schizophrenia, tumours, or cerebrovascular disease; (2) standard contraindications for MRI examinations; and (3) absence of a reliable informant.

### Neuropsychological test

Each participant underwent a standardized neuropsychological assessment battery. Global cognitive screening was performed using the MMSE, and disease severity was assessed using the CDR® and the 6 domains of CDR® plus the behaviour/comportment and language domains (CDR® plus NACC FTLD). The severity of behavioural abnormalities was assessed using the Frontal Behaviour Inventory (FBI), which can be separated into the negative apathy symptom subscale (first 12 items) and the positive disinhibition symptom subscale (last 12 items). Executive function was evaluated using the Trail Making Test (TMT) and the Stroop I and II tests. Language deficits were tested using the 30-item Boston Naming Test (BNT). Activities of living were assessed by the following scales: activities of daily living (ADL).

Controls in our plasma cohort did not complete the FBI, TMT, BNT and ADL scales. To clearly show how affected the patients truly are, we calculated the z scores by selecting another group of controls who were age- and sex-matched with bvFTD patients and completed the FBI, TMT, BNT and ADL scales.

### Inflammatory factor analysis

Peripheral blood was collected from each subject in acid-citrate dextrose Vacutainers (BD Biosciences; San Jose, Ca). Blood was processed to obtain plasma by centrifugation at 2100 rpm for 10 min, and plasma was harvested, aliquoted, and stored at − 80 °C until cytokine analysis. For Bio-Plex Pro Human Inflammation Panel (Bio-Rad, 17008653, Hercules, California, USA) detection, a panel of five cytokines (tumour necrosis superfamily member 13B [TNFSF/BAFF], IL-29/interferon [IFN]-λ2, IL-32, TWEAK/TNFSF12, and sCD30/TNFRSF8) were measured in the plasma. Samples were run at one time according to the manufacturer’s protocol, with one sample tested per subject. Briefly, 25 μl of sample was incubated with antibody-coupled fluorescent beads and then washed and incubated with biotinylated detection antibodies followed by streptavidin–phycoerythrin. The beads were then analysed using a flow based Luminex 100 suspension array system (Bioplex 200; Bio-Rad Laboratories, Inc.). Standard curves were generated by Bioplex Manager software to determine unknown sample concentrations, and reference cytokines were provided by the manufacturer in the kit. Unknown sample concentrations were below or above the standard curve detection range. The proinflammatory panel 1 (human) kit (MSD, K15396S, Rockville, USA) was then used to detect a panel of nine plasma cytokines (IFN-γ, IL-10, IL-12p70, IL-17A, IL-1β, IL-2, IL-4, IL-6, and TNF-α).

### Neuroimaging analysis

#### FDG–PET/MRI acquisition parameters

All images were acquired on a hybrid 3.0 T time-of-flight ^18^F-fluorodeoxyglucose (FDG)–PET/MRI scanner (SIGNA FDG–PET/MR, GE Healthcare, WI, USA). FDG–PET and MRI data were acquired simultaneously using a vendor-supplied 19-channel head and neck union coil. Three-dimensional (3D) T1-weighted sagittal images and FDG–PET volumes were acquired during the same session after administering 3.7 MBq/kg ^18^F-FDG.

The parameters of the T1 data were as follows: repetition time (TR) = 6.9 ms, echo time (TE) = 2.98 ms, flip angle = 12°, inversion time = 450 ms, matrix size = 256 × 256, field of view (FOV) = 256 × 256 mm^2^, slice thickness = 1 mm, 192 sagittal slices with no gap, voxel size = 1 × 1 × 1 mm^3^, and acquisition time = 4 min 48 s. Static ^18^F-FDG–PET data were acquired using the following parameters: matrix size = 192 × 192, FOV = 350 × 350 mm^2^, and pixel size = 1.82 × 1.82 × 2.78 mm^3^, including corrections for random coincidences, dead time, scatter, and photon attenuation. Attenuation correction was performed based on MRI of the brain (Atlas-based coregistration of two-point Dixon). The default attenuation correction sequence was automatically prescribed and acquired as follows: LAVA-Flex (GE Healthcare) axial acquisition, TR = 4 ms, TE = 1.7 ms, slice thickness = 5.2 mm with a 2.6 mm overlap, 120 slices, pixel size = 1.95 × 2.93 mm, and acquisition time = 18 s.

#### Structural image preprocessing

Data were preprocessed using the computational anatomy toolbox 12 (CAT12) (http://www.neuro.uni-jena.de/cat/), which is based on statistical parametric mapping 12 (SPM12). First, the DICOM files were converted into NIfTI format using MRIcron software (http://people.cas.sc.edu/rorden/mricron/index.html). Voxel-based morphometry preprocessing was performed using the default settings of the CAT12 toolbox and the “East Asian Brains” ICBM template. T1-weighted 3D images were segmented into grey matter (GM), white matter (WM), and cerebrospinal fluid (CSF) partitions. Subsequently, the GM and WM partitions of each subject in native space were high-dimensionally registered and normalized to the standard Montreal Neurological Institute space using diffeomorphic anatomical registration through exponentiated lie algebra normalization. The images were then smoothed using an 8-mm full-width half-maximum Gaussian kernel.

#### FDG–PET image preprocessing

FDG–PET images were preprocessed using SPM12, implemented in MATLAB (MathWorks, Natick, Massachusetts). After normalization of the structural MRI images, the transformation parameters determined by the T1-weighted image spatial normalization were applied to the coregistered FDG–PET images for FDG–PET spatial normalization. The images were then smoothed using an isotropic Gaussian kernel with an 8-mm full-width half-maximum. Finally, FDG–PET scan intensity was normalized using a whole cerebellum reference region to create standardized uptake value ratio (SUVR) images.

#### Regions of interest (ROI) analysis

We conducted atlas-based ROI analysis of the structural MRI and FDG–PET images to extract the regional GM volumes and SUVR of 90 brain regions from the automated anatomical labelling atlas for further partial correlation analysis.

### Statistical analysis

All statistical analyses were performed in SPSS Statistics Version 22 (IBM, Armonk, NK, USA). All tests were two-tailed with alpha set at *p* < 0.05. To compare differences between two groups, Student’s t test was used for normally distributed data, and the Mann‒Whitney U test was used for skewed data. ANOVA was used to compare the differences across three groups.

Partial correlation analysis was implemented using age and sex as covariates to explore the association between peripheral inflammatory markers, neuroimaging, and clinical measures.

Multivariable linear regression was used to identify the association between FDG–PET/MRI/clinical measures and the combination of cytokines. The false discovery rate (FDR) correction was used for the correlation analysis**.**

## Results

### Demographics and clinical data

Detailed demographic data and neuropsychological performance are summarized in Table 1. Thirty-nine patients with bvFTD were recruited, including 16 males and 23 females. There were no group differences in age, sex or years of education. Behaviour problems were prominent in bvFTD patients, which was shown by an FBI score of 24.44 ± 12.78, an apathy subscale score of 17.56 ± 8.39, and a disinhibition subscale score of 6.88 ± 5.58. Patients had poor neuropsychological performance for general mental status, shown by a mean MMSE score of < 24. The CDR® sum box score of patients was 8.36 ± 4.14, and the CDR® plus NACC FTLD sum box score was 10.72 ± 5.17. Executive functions were found to be impaired in patients: the TMT-A completion time was 117.56 ± 35.17 s, and the TMT-B completion time was 236.80 ± 81.01 s. Moreover, language deficits were found in patients with bvFTD with a BNT score of 17.77 ± 6.99.

**Table 1:** Participant demographic characteristics and neuropsychological score

	bvFTD (*n* = 39)	Control (*n* = 40)	*p* value
Age, years	62.31 ± 7.67	63.80 ± 6.16	0.34
Age at onset	60.03 ± 7.52	/	
Disease duration (m)	25.44 ± 16.24	/	
Sex (M/F)	16/23	15/25	0.75
Education, years	10.10 ± 3.64	11.16 ± 3.40	0.15
Cognitive status			
MMSE	16.77 ± 10.41	28.55 ± 0.90	< 0.001
Dementia rating			
CDR**®** SB	8.36 ± 4.14	0	< 0.001
CDR**®** global	1.69 ± 0.73	0	< 0.001
CDR® plus NACC FTLD SB	10.72 ± 5.17	0	< 0.001
BEHAV	1.74 ± 0.75	0	< 0.001
LANG	0.62 ± 0.56	0	< 0.001
Behavioural features			
FBI-total (*z* score)	24.44 ± 12.78 (0.82 ± 0.81)	/	/
FBI apathy (*z* score)	17.56 ± 8.39 (0.80 ± 0.85)	/	/
FBI disinhibition (*z* score)	6.88 ± 5.58 (0.67 ± 1.05)	/	/
Executive function			
TMT-A (*z* score)	117.56 ± 35.17 (0.75 ± 0.81)	/	/
TMT-B (*z* score)	236.80 ± 81.01 (0.79 ± 0.81)	/	/
Linguistics			
BNT (z score)	17.77 ± 6.99 (− 0.56 ± 1.09)	/	/
Activities of daily living			
ADL (z score)	33.76 ± 13.20 (0.64 ± 1.11)	/	/

*bvFTD* behavioural variant frontotemporal dementia, *MMSE* Mini-Mental State Examination, *CDR® global* standard 6 item CDR® global rating, *CDR®SB* sum of the boxes score of the 6 domains of the CDR, *CDR® plus NACC FTLD-SB* sum of the boxes score of the 6 domains of the CDR® plus the behaviour/comportment and language domains, *BEHAV* behaviour, *LANG* language, *TMT* Trail Making Test, *FBI* Frontal Behaviour Inventory, *BNT* Boston Naming Test. *ADL* activities of daily living scale; FBI, TMT, BNT and ADL are shown in *z* scores

### Group comparison of inflammatory factors

**Fig. 1:**
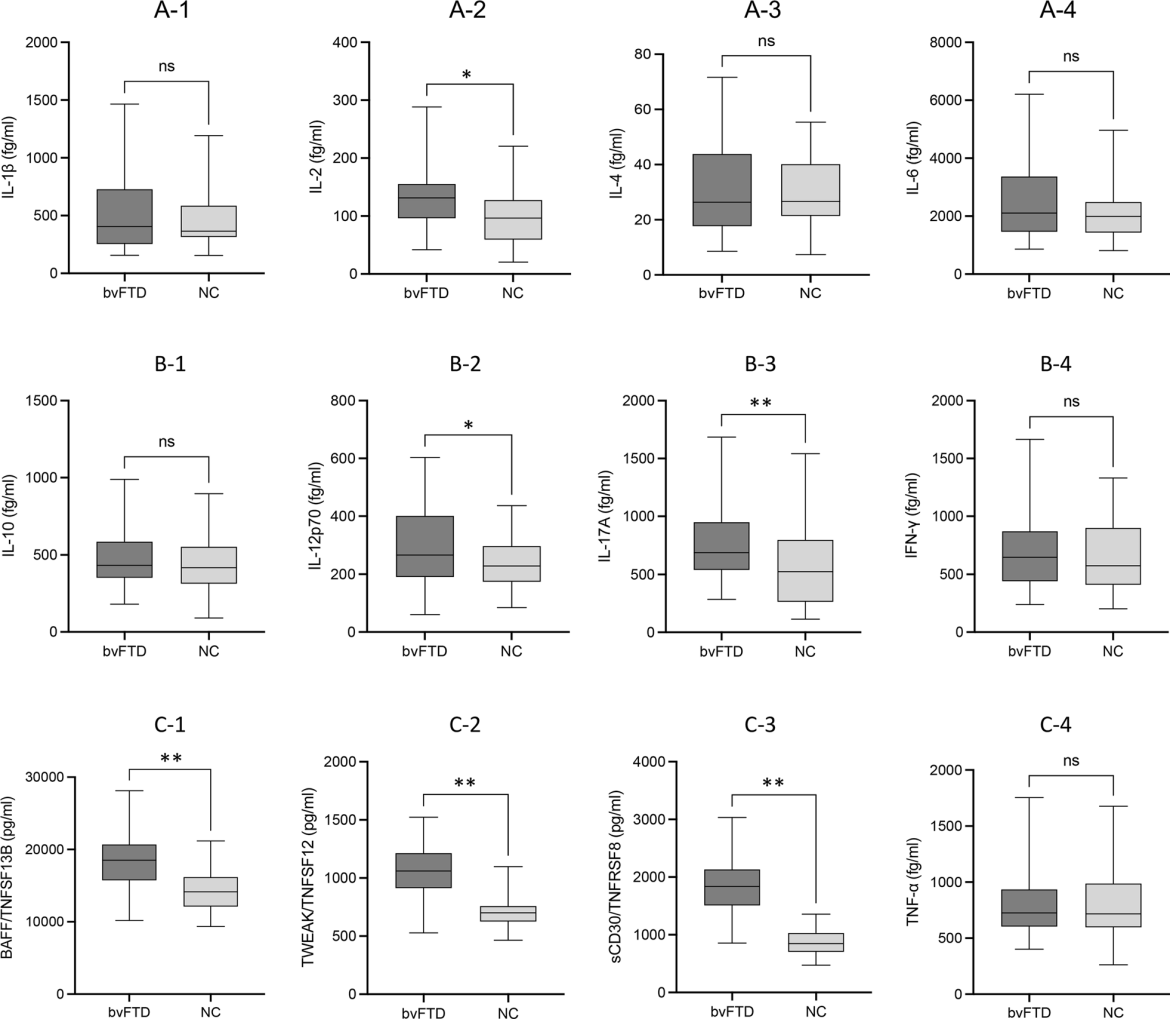
Comparison of inflammatory cytokine levels between groups. Compared with controls, levels of IL-2 (A-2), IL-12p70 (B-2), IL-17A (B-3), BAFF/TNFSF (C-1), TWEAK/TNFSF12 (C-2), and sCD30/TNFRSF8 (C-3) were increased in bvFTD patients. No group difference was found for IL-1β (A-1), IL-4 (A-3), IL-6 (A-4), IL-10 (B-1), IFN-γ (B-4), or TNF-α (C-4). * p < 0.05, ** < 0.001

IL-29/IFN λ2 and IL-32 were undetectable due to low concentrations. Among the remaining 12 inflammatory factors, six factors were increased in the bvFTD group, including IL-2 (fg/ml, 128.119 ± 49.015 vs. 97.092 ± 48.909, *p* = 0.006), IL-12p70 (fg/ml, 293.723 ± 136.569 vs. 238.932 ± 89.168, *p* = 0.038), IL-17A (fg/ml, 782.174 ± 329.605 vs. 533.462 ± 326.141, *p* < 0.001), BAFF/TNFSF (pg/ml, 18,319.351 ± 4105.147 vs. 114,380.479 ± 2700.754, *p* < 0.001), TWEAK/TNFSF12 (pg/ml, 1060.374 ± 217.316 vs. 704.581 ± 141.874, *p* < 0.001), and sCD30/TNFRSF8 (pg/ml, 1885.468 ± 871.310 vs. 871.310 ± 215.347, *p* < 0.001) (Fig. 1).

However, the other six inflammatory factors showed no between-group difference, including IL-1β (fg/ml, 558.850 ± 395.389 vs. 442.339 ± 210.883, *p* = 0.105), IL-4 (fg/ml, 30.254 ± 12.756 vs. 31.735 ± 17.019, *p* = 0.662), IL-6 (fg/ml,2603.620 ± 1446.585 vs. 2160.742 ± 978.452, *p* = 0.114), IL-10 (fg/ml, 479.757 ± 188.258 vs. 479.757 ± 188.258, *p* = 0.339), IFN-γ (fg/ml, 732.633 ± 387.201 vs. 633.485 ± 298.906, *p* = 206), and TNF-α (fg/ml, 777.467 ± 270.867 vs. 794.684 ± 324.308, *p* = 0.799) (Fig. 1).

### Subgroup comparison of inflammatory factors

We performed a further subgroup analysis and divided the patients with bvFTD into subgroups according to sex (male vs. female), age at onset (< 65 years vs. ≥ 65 years), disease duration (< 1 year vs. ≥ 1 year), with or without apathy, with or without disinhibition, FTLD–CDR® global (FTLD–CDR = 1, 2, or 3), FTLD-CDR® behaviour (1,2,3), and FTLD-language (with/without language deficit). Detailed information about the subgroup analysis is shown in Additional file 1: Tables S1–S8.

Female patients with bvFTD had a higher level of IL-12p70 (246.67 ± 79.48 vs. 333.37 ± 149.85, p = 0.0416). Patients with FTD who had a longer disease duration had increased proinflammatory factor levels (IL-2, 110.69 ± 44.29 vs. 208.64 ± 389.87, p = 0.0262) and decreased anti-inflammatory factor levels (IL-4, 42.46 ± 19.78 vs. 28.77 ± 14.46, p = 0.0219). Patients with disinhibition had a higher level of the proinflammatory markers IL-12p70 (359.33 ± 150.19 vs. 255.01 ± 99.69, p = 0.0129) and IL-6 (3775.78 ± 2987.84 vs. 1973.68 ± 925.48, p = 0.0255). Patients with FTD who had a severe disease status had a higher level of the proinflammatory factors IL-17A (532.27 ± 303.87 vs. 821.53 ± 441.92 vs. 2277.41 ± 3518.86, p = 0.0324) and IL-2 (124.38 ± 40.37 vs. 121.60 ± 44.52 vs. 499.43 ± 827.64, p = 0.0340). Patients with FTD who had a severe behavioural deficit had a higher level of the proinflammatory factor IL-17A (516.94 ± 305.97 vs. 766.01 ± 381.92 vs. 2184.30 ± 3221.69, p = 0.0290).

### The relationship between peripheral inflammation and central brain degeneration

**Fig. 2:**
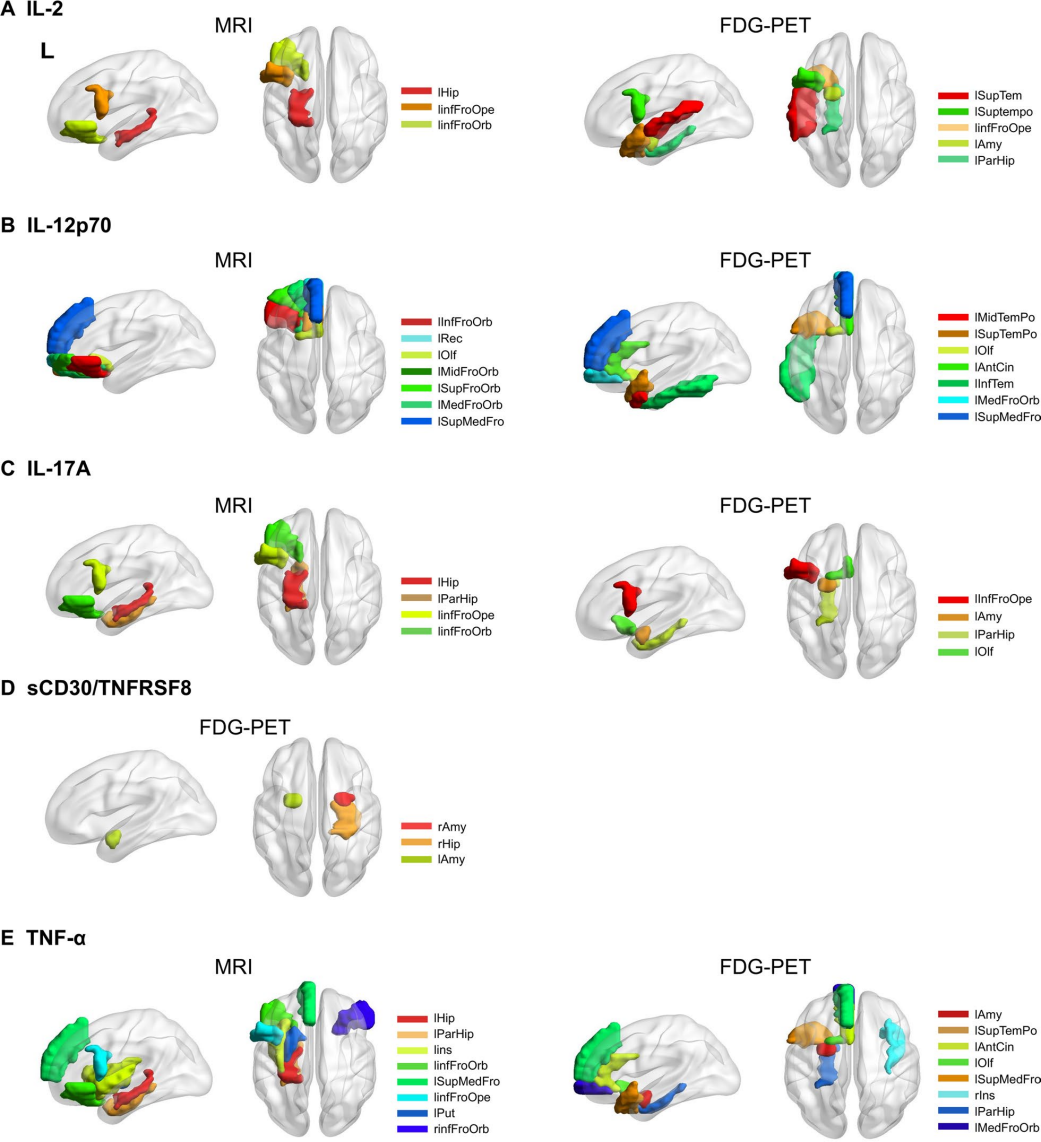
Brain regions associated with peripheral inflammation markers. Graph depiction of the brain regions significantly correlated with peripheral inflammation markers, including **A** IL2; **B** IL-12p70; **C** IL-17A; **D** sCD30/TNFRSF8; and **E** TNF-α. The brain regions are mainly distributed in frontal–temporal–limbic areas. The significant brain regions are demonstrated on a 3D brain template using the Brain-Net viewer toolbox. Detailed information about the brain regions is shown in Table 2. *L* left, *R* right, *InfFroOrb* inferior frontal orbital, *MidFroOrb* middle frontal orbital, *SupFroOrb* superior frontal orbital, *MedFroOrb* medial frontal orbital, *Rec* rectus, *Olf* olfactory, *SupMedFro* superior medial frontal, *InfFroOpe* inferior frontal operculum, *AntCin* anterior cingulate, *MidTemPo* middle temporal pole, *SupTemPo* superior temporal pole, *InfTem* inferior temporal gyrus, *SupTem* superior temporal gyrus, *Hip* hippocampus, *ParHip* para-hippocampus, *Ins* insula, *Put* putamen, *Amy* amygdala

The detailed results of the correlation analysis between cytokine levels and PET/MRI images are shown in Table 2 and Fig. 2. IL-2 levels were negatively correlated with atrophy of the hippocampus and frontal cortex and with hypometabolism of the temporal pole, frontal, striatal, and limbic cortex. IL-12p70 levels were negatively correlated with atrophy of the frontal cortex and with hypometabolism of the frontal, anterior cingulate, and temporal cortex. IL-17A levels were negatively correlated with atrophy of the frontal and limbic brain regions and with hypometabolism of the frontal, striatal and limbic brain regions. sCD30/TNFRSF8 levels were negatively correlated with hypometabolism of the amygdala and hippocampus. TNF-α levels were negatively correlated with atrophy of the frontal and striatal brain regions and with the hypometabolism of the frontal, striatal, temporal, and limbic regions.

**Table 2:** Partial correlation analysis between inflammatory cytokines and grey matter volume and metabolism

Inflammatory factors	Structural MRI	*r*/rho	FDR adjusted *P* value	FDG–PET	*r*/rho	FDR adjusted *P* value
IL-2	Left hippocampus	− 0.5153	0.0053	Left superior temporal	− 0.6110	0.0300
	Left inferior frontal gyrus, opercular part	− 0.4482	0.0175	Left superior temporal pole	− 0.5786	0.0435
	Left inferior frontal orbital	− 0.4100	0.0316	Left amygdala	− 0.5703	0.0061
				Left inferior frontal gyrus, opercular part	− 0.5581	0.0133
				Left para-hippocampus	− 0.4930	0.0161
IL-12p70	Left inferior frontal orbital	− 0.5769	0.0014	Left middle temporal pole	− 0.5878	0.0035
	Left rectus	− 0.5317	0.0038	Left superior temporal pole	− 0.5809	0.0008
	Left olfactory	− 0.5317	0.0038	Left olfactory	− 0.5768	0.0218
	Left middle frontal orbital	− 0.5288	0.0041	Left anterior cingulate	− 0.5228	0.0090
	Left superior frontal orbital	− 0.5098	0.0059	Left inferior temporal	− 0.5055	0.0277
	Left medial frontal orbital	− 0.5054	0.0065	Left medial frontal orbital	− 0.4691	0.0203
	Left superior medial frontal	− 0.5020	0.0069	Left superior medial frontal	− 0.4647	0.0218
IL-17A	Left hippocampus	− 0.6302	0.0004	Left inferior frontal gyrus, opercular part	− 0.6165	0.0357
	Left para-hippocampus	− 0.4900	0.0085	Left amygdala	− 0.5820	0.0018
	Left inferior frontal gyrus, opercular part	− 0.4151	0.0293	Left para-hippocampus	− 0.5335	0.0023
	Left inferior frontal orbital	− 0.4046	0.0342	Left olfactory	− 0.5112	0.0201
sCD30/TNFRSF8	ns			Right amygdala	− 0.5379	0.0264
				Right hippocampus	− 0.5325	0.0075
				Left amygdala	− 0.4860	0.0027
TNF-α	Left hippocampus	− 0.6471	0.0002	Left amygdala	− 0.5622	0.0021
	Left para-hippocampus	− 0.5401	0.0032	Left superior temporal pole	− 0.5544	0.0055
	Left insula	− 0.5162	0.0052	Left anterior cingulate	− 0.5077	0.0090
	Left inferior frontal orbital	− 0.5055	0.0065	Left olfactory	− 0.4928	0.0010
	Left superior medial frontal	− 0.5040	0.0066	Left superior medial frontal	− 0.4913	0.0154
	Left inferior frontal gyrus, opercular part	− 0.4956	0.0077	Right insula	− 0.4850	0.0267
	Left putamen	− 0.4879	0.0089	Left para-hippocampus	− 0.4823	0.0022
	Right inferior frontal orbital	− 0.4837	0.0095	Left medial frontal orbital	− 0.4499	0.0185

Adjusted for age, sex, and education. All results demonstrated in the table passed the false discovery rate (FDR) correction

### Association between peripheral inflammation and clinical measures

Some clinical measures were found to be significantly associated with inflammatory cytokines (uncorrected *p* < 0.05), and the detailed r/rho and p values are shown in Table 3. Plasma levels of BAFF/TNFSF13B were negatively correlated with MMSE scores and positively correlated with FBI apathy and FBI total scores. IL-4 levels were negatively correlated with disease duration. IL-6 levels were positively correlated with TMT-A and CDR® plus NACC FTLD BEHAV. IL-17A levels were positively associated with disease severity assessed by CDR® global and CDR® plus NACC FTLD BEHAV. TNF-α levels were positively correlated with CDR® SB, behaviour and language subdomains and CDR® plus NACC FTLD SB. After FDR correction, a significant association (FDR corrected *p* < 0.05) between BAFF/TNFSF13B levels and FBI apathy still existed.

**Table 3:** Correlation analysis between plasma levels of cytokines and neuropsychological scales

Cytokines	Scale	*r*/rho	Uncorrected *P* value	FDR adjusted *p* value
BAFF/TNFSF13B	MMSE	− 0.3558	0.0422	0.2277
	FBI Apathy	0.5085	0.0005	0.0145
	FBI Total	0.4343	0.0116	0.0962
IL-4	Disease duration	− 0.3090	0.0802	0.3192
IL-6	TMT-A	0.3507	0.0454	0.2349
	CDR® plus NACC FTLD BEHAV	0.3288	0.0442	0.2319
IL-17A	CDR® global	0.4319	0.0121	0.0972
	CDR® plus NACC FTLD BEHAV	0.4272	0.0132	0.1037
TNF-α	CDR® SB	0.3613	0.0389	0.2277
	CDR® plus NACC FTLD BEHAV	0.4336	0.0117	0.0962
	CDR® plus NACC FTLD LANG	0.3473	0.0477	0.2403
	CDR® plus NACC FTLD SB	0.3840	0.0274	0.1808

*MMSE* Mini-Mental State Examination, *CDR® global* standard 6 item CDR® global rating, *CDR®SB* sum of the boxes scores of the 6 domains of the CDR, *CDR® plus NACC FTLD-SB* sum of the boxes scores of the 6 domains of the CDR® plus the behaviour/comportment and language domains, *BEHAV* behaviour, *LANG* language, *TMT* Trail Making Test, *FBI* Frontal Behaviour Inventory, *BNT* Boston Naming Test

### Association between the combination of cytokines and central neurodegeneration

Structural MRI analysis (Additional file 1: Tables S9–S14) showed that the models of grey matter volume of the left hippocampus, left para-hippocampus, left amygdala, left fusiform, left superior temporal pole, left middle temporal gyrus, left middle temporal pole, and left inferior temporal gyrus were significant (*P* < 0.05). Grey matter volume of the **left hippocampus** (*R*^2^ = 0.6858, *p* = 0.0168) was negatively associated with the levels of IL-17A (*β* = − 1.1783, *p* = 0.0315) and TNF-α (*β* = − 0.6094, *p* = 0.0391). Grey matter volume of the **left para-hippocampus** (*R*^2^ = 0.6503, *p* = 0.0335) was negatively associated with the levels of TNF-α (*β* = − 0.6700, *p* = 0.324). Grey matter volume of the **left amygdala** (*R*^2^ = 0.6369, *p* = 0.0424**)** was negatively associated with the levels of TNF-α (*β* = − 0.7060, *p* = 0.276). Grey matter volume of the **left superior temporal pole** (*R*^2^ = 0.6384, *p* = 0.0413) was negatively associated with the levels of IL-10 (*β* = − 0.4700, *p* = 0.0470). Grey matter volume of the **left middle temporal gyrus** (*R*^2^ = 0.6677, *p* = 0.0242) was negatively associated with the levels of IL-10 (*β* = − 0.4814, *p* = 0.0352), IL-17A (*β* = − 1.2450, *p* = 0.0277), IL-1β (*β* = − 0.5151, *p* = 0.0067), IL-6 (*β* = − 0.3866, p = 0.0481) and TNF-α (*β* = − 0.6210, *p* = 0.0407). Grey matter volume of the left **inferior temporal gyrus** (*R*^2^ = 0.7063, *p* = 0.0107) was negatively associated with the levels of IL-17A (*β* = − 1.2855, *p* = 0.0170), IL-1β (*β* = − 0.4483, *p* = 0.0109) and IL-6 (*β* = − 0.4131, *p* = 0.0270).

FDG–PET analysis (Additional file 1: Table S15–S19) showed that models of grey matter metabolism of the left olfactory gyrus, left hippocampus, left para-hippocampus, left amygdala, left middle temporal pole, and left inferior temporal gyrus were significant (*p* < 0.05). Grey matter metabolism of the **left olfactory gyrus** (*R*^2^ = 0.7245, *p* = 0.0045) was negatively associated with the levels of TWEAK/TNFSF12 (*β* = − 0.3964, *p* = 0.0254) and IL-17A (*β* = − 0.9806, *p* = 0.0372). Grey matter metabolism in the left **para-hippocampus** (*R*^2^ = 0.7919, *p* < 0.001) was negatively associated with the levels of TWEAK/TNFSF12 (*β* = − 0.4195, *p* = 0.0082) and IFN-γ (*β* = − 0.3740, *p* = 0.0269). Grey matter metabolism of the **left amygdala** (*R*^2^ = 0.6998, *p* = 0.0083) was negatively associated with the levels of sCD30/TNFRSF8 (*β* = − 0.4352, *p* = 0.0285). Grey matter metabolism of the **left middle temporal pole** (*R*^2^ = 0.6575, *p* = 0.0210) was negatively associated with the levels of TWEAK/TNFSF12 (*β* = − 0.3814, *p* = 0.497) and IL-12p70 (*β* = − 0.4239, *p* = 0.0491). Grey matter metabolism of the **left inferior temporal gyrus** (*R*^2^ = 0.6509, *p* = 0.0239) was negatively associated with the levels of BAFF/TNFSF13B (*β* = − 0.3827, *p* = 0.0305) and TWEAK/TNFSF12 (*β* = − 0.4181, *p* = 0.347).

### Association between the combination of cytokines and clinical measures

The detailed data are listed in Additional file 1: Tables S20–S25. Levels of IL-17A (*β* = 0.9285, *p* = 0.0284) were positively correlated with CDR® plus NACC FTLD sum of box (*R*^2^ = 1563, *p* = 0.0469). Levels of IL-17A (*β* = 0.3472, *p* = 0.0378) were positively correlated with CDR® sum of box (*R*^2^ = 0.2092, *p* = 0.0397). Levels of IL-17A (*β* = − 0.9305, *p* = 0.0327) and IL-1β (*β* = − 0.3351, *p* = 0.0323) were negatively correlated with BNT (*R*^2^ = 0.3140, *p* = 0.0467). Levels of BAFF/TNFSF13B (*β* = 0.5269, *p* < 0.001) were positively correlated with FBI apathy scores (*R*^2^ = 0.3872, *p* = 0.0048). Levels of BAFF/TNFSF13B (*β* = 0.3703, *p* = 0.0211) were positively correlated with FBI total (*R*^2^ = 0.1613, *p* = 0.0422). Levels of IL-4 (β = − 0.3646, *p* = 0.0228) were negatively correlated with the disease duration (*R*^2^ = 0.1667, *p* = 0.0375).

## Discussion

This study found abnormal levels of markers of peripheral inflammation in patients with bvFTD, which correlated with brain degeneration and clinical measures. The strength of this study is that it is the first to explore the association between peripheral inflammation and brain metabolism in patients with bvFTD, confirming their association. Our study provides new insight into peripheral inflammation factors that might be putative markers associated with central neurodegeneration contributing to the pathophysiology of FTD. However, as we did not directly investigate the association between central and peripheral inflammation, our exploratory findings should be interpreted with caution.

In frontotemporal dementia, the glial phenotypic landscape is altered in the frontotemporal cortex, with microglial activation/dystrophy and astrogliosis. Then, dysregulated secretion of cytokines occurs, with an increased proinflammatory response and impaired anti-inflammatory response, causing neuronal death and neurodegeneration [12]. With the impairment of the blood–brain barrier, dysregulated pro- and anti-inflammatory factors are observed in both CSF and serum [12, 19]. Based on previous literature, there is a hypothesis that inflammation occurs in FTD patients with increased levels of proinflammatory markers (IL-1β, IL-2, IL-6, IL-12p70, IL-17A, IFN-γ, TNF-α, BAFF/TNFSF13B, TWEAK/TNFSF12, and sCD30/TNFRSF8) and decreased levels of anti-inflammatory markers (IL-4 and IL-10). However, in our study, we found that the levels of some proinflammatory markers were increased, including IL-2, IL-12p70, IL-17A, BAFF/TNFSF13B, TWEAK/TNFSF12, and sCD30/TNFRSF8; the other six proinflammatory markers and two anti-inflammatory markers were not significantly different between groups, which might be because of the heterogeneity and small sample size of the patient group.

Serum and CSF levels of ILs have frequently been investigated in previous studies. In this study, IL-2, IL-12p70, and IL-17A were increased in the bvFTD group. IL-17A is increased in C9 murine models, which supports our clinical findings [20, 21]. However, in a previous clinical study, IL-17A and two other factors, IL-2 and IL-12p70, were undetectable or reduced compared with controls [7, 22, 23]. IL-6 levels were reported to be elevated in previous studies on FTD [6, 24–26]; however, they were normal in our study, which might be due to the different disease phases or sample testing methods. IFN-γ, IL-1β, IL-4, and IL-10 levels have previously been reported to be normal in studies targeting genetic or sporadic FTD, which was consistent with our results [7, 22, 23]. The TNF superfamily is one of the most evaluated cytokine families in inflammatory responses in neurodegenerative diseases and is often reported to be abnormal in disease conditions [6, 15, 22, 23, 27–30]. Animal studies have also demonstrated a direct association between progranulin and microglial TNF production [31]. In this study, we also found increased levels of BAFF/TNFSF13B, TWEAK/TNFSF12, and sCD30/TNFRSF8 in bvFTD; however, TNF-α was not changed, which is consistent with some studies [6, 29] and inconsistent with others [23, 27, 28].

Five factors were found to be significantly associated with central degeneration, including IL-2, IL-12p70, IL-17A, sCD30/TNFRSF8, and TNF-α. In summary, these proteins were associated with brain atrophy mainly distributed in the frontal–limbic–striatal brain regions and brain metabolism mainly in the frontal–temporal–limbic–striatal regions. The associated atrophy pattern is consistent with the typical FTD-related atrophy pattern [32]. Hypometabolic regions are more widespread than atrophy in the frontal, temporal, limbic, and subcortical nuclei, which is consistent with the disruption of the neurocircuitry of the cortical–limbic–striatal brain regions, constitute the salience network in FTD [33–35]. [^18^F]-FDG is a radiotracer used to measure the local cerebral metabolic rate of glucose, which is indicative of synaptic activity in the brain. [^18^F]-FDG–PET is used to visualize the distribution of neural injury and genuine synaptic dysfunction, which is not only due to local neural damage but also remote from morphological damage in other components of the functional circuit [36, 37]. All these associations provide evidence that nonspecific inflammatory markers contribute to disease-specific brain neural damage.

Increased immune cell activation in the context of sporadic FTD could lead to the increased release of inflammatory factors, exacerbating neuronal damage through excessive phagocytosis in key functional brain regions and causing functional changes and clinical deficit [12]. In our study, a tendency was found that inflammatory cytokines correlated with clinical measures, including general status, behaviour deficit, executive dysfunction, and disease severity. Notably, BAFF/TNFSF13B was correlated with apathy after stringent correction, a key symptom of bvFTD, suggesting the significant role of the TNF family in disease pathogenesis. In other studies, some cytokines were also reported to be correlated with neuropsychological scales such as MMSE or ADCS–ADL decline rate in FTD [7, 38]. In addition, neuroinflammation was affected by age and disease duration. In this study, we found a tendency towards a longer disease duration with increased proinflammatory marker (IL-2) levels and decreased anti-inflammatory factor (IL-4) levels in the subgroup analysis, and IL-4 levels were negatively associated with disease duration. However, we did not find any associations between cytokines and age. In other studies, levels of inflammatory factors such as serum chemokine ligand 4 (CCL4) were positively correlated with age[23], and CSF soluble triggering receptor expressed on myeloid cells 2 (sTREM2) levels were positively correlated with age and negatively correlated with disease duration[39].

18 KDa translocator protein (TSPO) expressed in glia, including microglia and astrocytes, was reported to be quantified using TSPO ligands in noninvasive PET examination[40, 41]. Overexpression of TSPO in active microglia and astrocytes was confirmed to be a reliable indicator of neuroinflammation in other neurodegenerative diseases [42]. Study in FTD using the TSPO ligand ^11^C-PK1119 was reported, which labelled the presence of an active glial response in frontotemporal brain regions, providing evidence for neuroinflammation in disease-specific brain regions[43]. Increased expression of TSPO was often considered associated with upregulated proinflammatory markers[12]. However, in FTD patients, the associations between in vivo TSPO PET and central/peripheral inflammatory markers remain elusive and can be investigated in future research.

This study has several limitations, and our results should be interpreted with caution. First, the sample size was relatively small due to the challenges in enrolling patients who completed both the inflammatory factor test and hybrid FDG–PET/MRI. Second, the cross-sectional design and correlation analysis cannot explain the causality of the direction. Third, our patients were all clinically diagnosed with probable bvFTD without pathological verification. Further molecular imaging, such as tau PET or post-mortem examination, which can reflect pathological alterations, should be conducted. Finally, direct correlation analysis between peripheral and central inflammation was not studied because only peripheral markers from the plasma were collected.

## Conclusions

The levels of peripheral inflammatory markers were found to be abnormal in patients with bvFTD, which correlated with disease-specific brain atrophy, hypometabolism, and main symptoms. These data will broaden our knowledge on how disturbances in immunity and inflammation participate in disease-specific brain damage, identifying promising targets for diagnosis, further treatment, and monitoring therapeutic efficacy.

## Supplementary Information

**Additional file 1: Table S1.** Group comparison of plasma levels of inflammatory cytokines between male and female. **Table S2.** Group comparison of plasma levels of inflammatory cytokines between Age at sample <65years and ≥65years. **Table S3.** Group comparison of plasma levels of inflammatory cytokines between disease duration <1 year and ≥1 year. **Table S4.** Group comparison of plasma levels of inflammatory cytokines between patients with/without apathy. **Table S5.** Group comparison of plasma levels of inflammatory cytokines between patients with/without disinhibition. **Table S6.** Group comparison of plasma levels of inflammatory cytokines in different disease severity. **Table S7.** Group comparison of plasma levels of inflammatory cytokines in different severity of behavioral deficit. **Table S8.** Group comparison of plasma levels of inflammatory cytokines between patients with/without language deficit. **Table S9.** Multiple regression analysis for associations between grey matter volume of left hippocampus and plasma levels of cytokines in FTD group. **Table S10.** Multiple regression analysis for associations between grey matter volume of left para-hippocampus and plasma levels of cytokines in FTD group. **Table S11.** Multiple regression analysis for associations between grey matter volume of left amygdala and plasma levels of cytokines in FTD group. **Table S12.** Multiple regression analysis for associations between grey matter volume of left superior temporal pole and plasma levels of cytokines in FTD group. **Table S13.** Multiple regression analysis for associations between grey matter volume of left middle temporal gyrus and plasma levels of cytokines in FTD group. **Table S14.** Multiple regression analysis for associations between grey matter volume of left inferior temporal gyrus and plasma levels of cytokines in FTD group. **Table S15.** Multiple regression analysis for associations between grey matter metabolism of left olfactory gyrus and plasma levels of cytokines in FTD group. **Table S16.** Multiple regression analysis for associations between grey matter metabolism of left para-hippocampus and plasma levels of cytokines in FTD group. **Table S17.** Multiple regression analysis for associations between grey matter metabolism of left amygdala and plasma levels of cytokines in FTD group. **Table S18.** Multiple regression analysis for associations between grey matter metabolism of left middle temporal pole and plasma levels of cytokines in FTD group. **Table S19.** Multiple regression analysis for associations between grey matter metabolism of left inferior temporal gyrus and plasma levels of cytokines in FTD group. **Table S20.** Multiple regression analysis for associations between CDR® plus NACC FTLD sum of box and plasma levels of cytokines in FTD group. **Table S21.** Multiple regression analysis for associations between CDR® sum of box and plasma levels of cytokines in FTD group. **Table S22.** Multiple regression analysis for associations between BNT and plasma levels of cytokines in FTD group. **Table S23.** Multiple regression analysis for associations between FBI apathy scores and plasma levels of cytokines in FTD group. **Table S24.** Multiple regression analysis for associations between FBI total score and plasma levels of cytokines in FTD group. **Table S25.** Multiple regression analysis for associations between disease duration and plasma levels of cytokines in FTD group.

## Data Availability

The data sets used and analysed during the current study are available from the corresponding author on reasonable request.

## Abbreviations

FTD: Frontotemporal dementia
bvFTD: Behavioural variant frontotemporal dementia
MMSE: Mini-Mental State Examination
FTLD: Frontotemporal lobar degeneration
CDR: Clinical dementia rating scale
TMT: Trail Making Test
FBI: Frontal Behaviour Inventory
BNT: Boston Naming Test
ADL: Activities of daily living scale
IADL: Instrumental activities of daily living subscale
BADL: Basic activities of daily living subscale
IL: Interleukin
TNF: Tumour necrosis factor
IFN: Interferon
FDG–PET/MRI: Fluorodeoxyglucose–positron emission tomography/magnetic resonance imaging
GM: Grey matter
WM: White matter
CSF: Cerebrospinal fluid
SUVR: Standardized uptake value ratio
FDR: False discovery rate

## References

[1] Johnson JK, Diehl J, Mendez MF, Neuhaus J, Shapira JS, Forman M, Chute DJ, Roberson ED, Pace-Savitsky C, Neumann M (2005). Frontotemporal lobar degeneration: demographic characteristics of 353 patients. Arch Neurol.

[2] Boxer AL, Gold M, Huey E, Gao FB, Burton EA, Chow T, Kao A, Leavitt BR, Lamb B, Grether M (2013). Frontotemporal degeneration, the next therapeutic frontier: molecules and animal models for frontotemporal degeneration drug development. Alzheimers Dement.

[3] Filiano AJ, Martens LH, Young AH, Warmus BA, Zhou P, Diaz-Ramirez G, Jiao J, Zhang Z, Huang EJ, Gao FB (2013). Dissociation of frontotemporal dementia-related deficits and neuroinflammation in progranulin haploinsufficient mice. J Neurosci.

[4] O'Rourke JG, Bogdanik L, Yáñez A, Lall D, Wolf AJ, Muhammad AK, Ho R, Carmona S, Vit JP, Zarrow J (2016). C9orf72 is required for proper macrophage and microglial function in mice. Science.

[5] Bussian TJ, Aziz A, Meyer CF, Swenson BL, van Deursen JM, Baker DJ (2018). Clearance of senescent glial cells prevents tau-dependent pathology and cognitive decline. Nature.

[6] Bossù P, Salani F, Alberici A, Archetti S, Bellelli G, Galimberti D, Scarpini E, Spalletta G, Caltagirone C, Padovani A, Borroni B (2011). Loss of function mutations in the progranulin gene are related to pro-inflammatory cytokine dysregulation in frontotemporal lobar degeneration patients. J Neuroinflammation.

[7] Katisko K, Solje E, Korhonen P, Jääskeläinen O, Loppi S, Hartikainen P, Koivisto AM, Kontkanen A, Korhonen VE, Helisalmi S (2020). Peripheral inflammatory markers and clinical correlations in patients with frontotemporal lobar degeneration with and without the C9orf72 repeat expansion. J Neurol.

[8] Oeckl P, Weydt P, Steinacker P, Anderl-Straub S, Nordin F, Volk AE, Diehl-Schmid J, Andersen PM, Kornhuber J, Danek A (2019). Different neuroinflammatory profile in amyotrophic lateral sclerosis and frontotemporal dementia is linked to the clinical phase. J Neurol Neurosurg Psychiatry.

[9] Lant SB, Robinson AC, Thompson JC, Rollinson S, Pickering-Brown S, Snowden JS, Davidson YS, Gerhard A, Mann DM (2014). Patterns of microglial cell activation in frontotemporal lobar degeneration. Neuropathol Appl Neurobiol.

[10] Bellucci A, Bugiani O, Ghetti B, Spillantini MG (2011). Presence of reactive microglia and neuroinflammatory mediators in a case of frontotemporal dementia with P301S mutation. Neurodegener Dis.

[11] Taipa R, Brochado P, Robinson A, Reis I, Costa P, Mann DM, Melo Pires M, Sousa N (2017). Patterns of microglial cell activation in Alzheimer disease and frontotemporal lobar degeneration. Neurodegener Dis.

[12] Bright F, Werry E, Dobson-Stone C, Piguet O, Ittner L, Halliday G, Hodges J, Kiernan M, Loy C, Kassiou M, Kril J (2019). Neuroinflammation in frontotemporal dementia. Nat Rev Neurol.

[13] Ransohoff RM (2016). How neuroinflammation contributes to neurodegeneration. Science.

[14] Swift IJ, Sogorb-Esteve A, Heller C, Synofzik M, Otto M, Graff C, Galimberti D, Todd E, Heslegrave AJ, van der Ende EL (2021). Fluid biomarkers in frontotemporal dementia: past, present and future. J Neurol Neurosurg Psychiatry.

[15] Vieira ÉLM, Caramelli P, Rocha NP, Freitas Cardoso MDG, de Miranda AS, Teixeira AL, de Souza LC (2021). Tumor necrosis factor superfamily molecules are increased in behavioral variant frontotemporal dementia and correlate with cortical atrophy: an exploratory investigation. J Neuroimmunol.

[16] Deverman BE, Patterson PH (2009). Cytokines and CNS development. Neuron.

[17] Boulanger LM (2009). Immune proteins in brain development and synaptic plasticity. Neuron.

[18] Rascovsky K, Hodges J, Knopman D, Mendez M, Kramer J, Neuhaus J, van Swieten J, Seelaar H, Dopper E, Onyike C (2011). Sensitivity of revised diagnostic criteria for the behavioural variant of frontotemporal dementia. Brain.

[19] Gerrits E, Giannini LAA, Brouwer N, Melhem S, Seilhean D, Le Ber I, Kamermans A, Kooij G, de Vries HE, Boddeke E (2022). Neurovascular dysfunction in GRN-associated frontotemporal dementia identified by single-nucleus RNA sequencing of human cerebral cortex. Nat Neurosci.

[20] Atanasio A, Decman V, White D, Ramos M, Ikiz B, Lee HC, Siao CJ, Brydges S, LaRosa E, Bai Y (2016). C9orf72 ablation causes immune dysregulation characterized by leukocyte expansion, autoantibody production, and glomerulonephropathy in mice. Sci Rep.

[21] Burberry A, Suzuki N, Wang JY, Moccia R, Mordes DA, Stewart MH, Suzuki-Uematsu S, Ghosh S, Singh A, Merkle FT (2016). Loss-of-function mutations in the C9ORF72 mouse ortholog cause fatal autoimmune disease. Sci Transl Med.

[22] Galimberti D, Bonsi R, Fenoglio C, Serpente M, Cioffi SM, Fumagalli G, Arighi A, Ghezzi L, Arcaro M, Mercurio M (2015). Inflammatory molecules in Frontotemporal Dementia: cerebrospinal fluid signature of progranulin mutation carriers. Brain Behav Immun.

[23] Roos P, von Essen MR, Nielsen TT, Johannsen P, Stokholm J, Bie AS, Waldemar G, Simonsen AH, Heslegrave A, Zetterberg H (2018). Inflammatory markers of CHMP2B-mediated frontotemporal dementia. J Neuroimmunol.

[24] Gibbons L, Rollinson S, Thompson JC, Robinson A, Davidson YS, Richardson A, Neary D, Pickering-Brown SM, Snowden JS, Mann DM (2015). Plasma levels of progranulin and interleukin-6 in frontotemporal lobar degeneration. Neurobiol Aging.

[25] Galimberti D, Schoonenboom N, Scheltens P, Fenoglio C, Venturelli E, Pijnenburg YA, Bresolin N, Scarpini E (2006). Intrathecal chemokine levels in Alzheimer disease and frontotemporal lobar degeneration. Neurology.

[26] Galimberti D, Venturelli E, Fenoglio C, Guidi I, Villa C, Bergamaschini L, Cortini F, Scalabrini D, Baron P, Vergani C (2008). Intrathecal levels of IL-6, IL-11 and LIF in Alzheimer's disease and frontotemporal lobar degeneration. J Neurol.

[27] Sjögren M, Folkesson S, Blennow K, Tarkowski E (2004). Increased intrathecal inflammatory activity in frontotemporal dementia: pathophysiological implications. J Neurol Neurosurg Psychiatry.

[28] Miller ZA, Rankin KP, Graff-Radford NR, Takada LT, Sturm VE, Cleveland CM, Criswell LA, Jaeger PA, Stan T, Heggeli KA (2013). TDP-43 frontotemporal lobar degeneration and autoimmune disease. J Neurol Neurosurg Psychiatry.

[29] Fraga VG, Magalhães CA, Loures CMG, de Souza LC, Guimarães HC, Zauli DAG, Carvalho MDG, Ferreira CN, Caramelli P, de Sousa LP, Gomes KB (2019). Inflammatory and pro-resolving mediators in frontotemporal dementia and Alzheimer's disease. Neuroscience.

[30] Rainero I, Rubino E, Cappa G, Rota E, Valfrè W, Ferrero P, Fenoglio P, Baci D, D'Amico G, Vaula G (2009). Pro-inflammatory cytokine genes influence the clinical features of frontotemporal lobar degeneration. Dement Geriatr Cogn Disord.

[31] Krabbe G, Minami SS, Etchegaray JI, Taneja P, Djukic B, Davalos D, Le D, Lo I, Zhan L, Reichert MC (2017). Microglial NFκB-TNFα hyperactivation induces obsessive-compulsive behavior in mouse models of progranulin-deficient frontotemporal dementia. Proc Natl Acad Sci U S A.

[32] Chu M, Liu L, Wang J, Liu L, Kong Y, Jing D, Xie K, Cui Y, Cui B, Zhang J (2021). Investigating the roles of anterior cingulate in behavioral variant frontotemporal dementia: a PET/MRI study. J Alzheimers Dis.

[33] Liu L, Chu M, Nie B, Liu L, Xie K, Cui Y, Kong Y, Chen Z, Nan H, Chen K (2022). Reconfigured metabolism brain network in asymptomatic microtubule-associated protein tau mutation carriers: a graph theoretical analysis. Alzheimers Res Ther.

[34] Zhou J, Greicius MD, Gennatas ED, Growdon ME, Jang JY, Rabinovici GD, Kramer JH, Weiner M, Miller BL, Seeley WW (2010). Divergent network connectivity changes in behavioural variant frontotemporal dementia and Alzheimer's disease. Brain.

[35] Zhou J, Seeley WW (2014). Network dysfunction in Alzheimer's disease and frontotemporal dementia: implications for psychiatry. Biol Psychiatry.

[36] Pitel AL, Aupée AM, Chételat G, Mézenge F, Beaunieux H, de la Sayette V, Viader F, Baron JC, Eustache F, Desgranges B (2009). Morphological and glucose metabolism abnormalities in alcoholic Korsakoff's syndrome: group comparisons and individual analyses. PLoS ONE.

[37] Segobin S, La Joie R, Ritz L, Beaunieux H, Desgranges B, Chételat G, Pitel AL, Eustache F (2015). FDG-PET contributions to the pathophysiology of memory impairment. Neuropsychol Rev.

[38] Woollacott IOC, Swift IJ, Sogorb-Esteve A, Heller C, Knowles K, Bouzigues A, Russell LL, Peakman G, Greaves CV, Convery R (2022). CSF glial markers are elevated in a subset of patients with genetic frontotemporal dementia. Ann Clin Transl Neurol.

[39] Woollacott IOC, Nicholas JM, Heslegrave A, Heller C, Foiani MS, Dick KM, Russell LL, Paterson RW, Keshavan A, Fox NC (2018). Cerebrospinal fluid soluble TREM2 levels in frontotemporal dementia differ by genetic and pathological subgroup. Alzheimers Res Ther.

[40] Sandiego CM, Gallezot JD, Pittman B, Nabulsi N, Lim K, Lin SF, Matuskey D, Lee JY, O'Connor KC, Huang Y (2015). Imaging robust microglial activation after lipopolysaccharide administration in humans with PET. Proc Natl Acad Sci U S A.

[41] Luu TG, Kim HK (2022). (18)F-Radiolabeled Translocator Protein (TSPO) PET tracers: recent development of TSPO radioligands and their application to PET study. Pharmaceutics.

[42] Ching AS, Kuhnast B, Damont A, Roeda D, Tavitian B, Dollé F (2012). Current paradigm of the 18-kDa translocator protein (TSPO) as a molecular target for PET imaging in neuroinflammation and neurodegenerative diseases. Insights Imaging.

[43] Cagnin A, Rossor M, Sampson EL, Mackinnon T, Banati RB (2004). In vivo detection of microglial activation in frontotemporal dementia. Ann Neurol.

