# Supplementary material for: Peripheral inflammation in behavioural variant frontotemporal dementia: associations with central degeneration and clinical measures
Source: J Neuroinflammation. 2023 Mar 8;20:65. doi: 10.1186/s12974-023-02746-5 (PMC9996857; doi:10.1186/s12974-023-02746-5)
Supplement: Supplementary file 1 — Additional file 1: Table S1. Group comparison of plasma levels of inflammatory cytokines between male and female. Table S2. Group comparison of plasma levels of inflammatory cytokines between Age at sample <65years and ≥65years. Table S3. Group comparison of plasma levels of inflammatory cytokines between disease duration <1 year and ≥1 year. Table S4. Group comparison of plasma levels of inflammatory cytokines between patients with/without apathy. Table S5. Group comparison of plasma levels of inflammatory cytokines between patients with/without disinhibition. Table S6. Group comparison of plasma levels of inflammatory cytokines in different disease severity. Table S7. Group comparison of plasma levels of inflammatory cytokines in different severity of behavioral deficit. Table S8. Group comparison of plasma levels of inflammatory cytokines between patients with/without language deficit. Table S9. Multiple regression analysis for associations between grey matter volume of left hippocampus and plasma levels of cytokines in FTD group. Table S10. Multiple regression analysis for associations between grey matter volume of left para-hippocampus and plasma levels of cytokines in FTD group. Table S11. Multiple regression analysis for associations between grey matter volume of left amygdala and plasma levels of cytokines in FTD group. Table S12. Multiple regression analysis for associations between grey matter volume of left superior temporal pole and plasma levels of cytokines in FTD group. Table S13. Multiple regression analysis for associations between grey matter volume of left middle temporal gyrus and plasma levels of cytokines in FTD group. Table S14. Multiple regression analysis for associations between grey matter volume of left inferior temporal gyrus and plasma levels of cytokines in FTD group. Table S15. Multiple regression analysis for associations between grey matter metabolism of left olfactory gyrus and plasma levels of cytokines in FTD group. Table S16. Mu [file 12974_2023_2746_MOESM1_ESM.docx]

**Table S1 Group comparison of** **plasma levels of inflammatory cytokines between male and female**

| Cytokine | Male (n=16) | Female (n=23) | p-value |
| --- | --- | --- | --- |
| BAFF/TNFSF13B | 20011.54±2338.31 | 18881.48±8211.02 | 0.5967 |
| TWEAK/TNFSF12 | 1117.09±263.99 | 1020.92±173.46 | 0.1774 |
| sCD30/TNFRSF8 | 2023.26±382.50 | 1789.61±513.73 | 0.1312 |
| IFN-γ | 581.20±339.74 | 981.89±869.98 | 0.0891 |
| IL-10 | 544.51±313.99 | 521.97±303.39 | 0.8232 |
| IL-12p70 | **246.67±79.48** | **333.37±149.85** | **0.0416*** |
| IL-17A | 576.78±386.70 | 1145.21±1844.25 | 0.2340 |
| IL-1β | 2343.22±7282.48 | 609.67±431.04 | 0.2594 |
| IL-2 | 144.21±43.74 | 206.61±433.48 | 0.5711 |
| IL-4 | 29.79±15.34 | 34.61±18.21 | 0.3921 |
| IL-6 | 3286.32±2464.21 | 2862.64±2585.90 | 0.6110 |
| TNF-α | 716.83±171.88 | 830.01±312.72 | 0.1973 |

**Table S2 Group comparison of plasma levels of inflammatory cytokines between Age at sample <65years and ≥65years.**

| Cytokine | Age at sample <65years (n=27) | Age at sample≥65years (n=12) | p-value |
| --- | --- | --- | --- |
| BAFF/TNFSF13B | 18095.00±3614.37 | 22157.81±10002.44 | 0.0684 |
| TWEAK/TNFSF12 | 1081.69±221.04 | 1012.42±209.86 | 0.3652 |
| sCD30/TNFRSF8 | 1859.47±441.74 | 1943.97±554.58 | 0.6135 |
| IFN-γ | 883.33±826.56 | 669.40±399.30 | 0.4013 |
| IL-10 | 547.94±326.68 | 493.58±253.93 | 0.6127 |
| IL-12p70 | 322.53±135.22 | 242.18±109.01 | 0.0785 |
| IL-17A | 927.24±1676.66 | 877.73±798.21 | 0.9233 |
| IL-1β | 1726.03±5590.26 | 409.26±306.41 | 0.4235 |
| IL-2 | 202.59±398.83 | 132.46±45.57 | 0.5502 |
| IL-4 | 33.17±15.85 | 31.42±20.19 | 0.7719 |
| IL-6 | 2799.81±2171.76 | 3568.91±3195.81 | 0.3847 |
| TNF-α | 787.07±276.19 | 775.72±257.81 | 0.9046 |

**Table S3 Group comparison of plasma levels of inflammatory cytokines between disease duration <1 year and ≥1 year.**

| Cytokine | Disease duration <1 year  (n=11) | Disease duration ≥1 year (n=28) | p-value |
| --- | --- | --- | --- |
| BAFF/TNFSF13B | 20663.06±11120.27 | 18827.32±3410.79 | 0.4306 |
| TWEAK/TNFSF12 | 1095.12±170.40 | 1046.72±234.58 | 0.5386 |
| sCD30/TNFRSF8 | 1895.85±618.41 | 1881.39±416.78 | 0.9329 |
| IFN-γ | 1126.88±1148.92 | 695.97±440.15 | 0.0946 |
| IL-10 | 585.59±299.73 | 509.85±308.21 | 0.4909 |
| IL-12p70 | 300.94±133.03 | 296.57±133.69 | 0.9272 |
| IL-17A | 690.46±482.63 | 999.04±1689.16 | 0.5574 |
| IL-1β | 748.02±527.79 | 1545.92±5511.43 | 0.6373 |
| IL-2 | **110.69±44.29** | **208.64±389.87** | **0.0262*** |
| IL-4 | **42.46±19.78** | **28.77±14.46** | **0.0219*** |
| IL-6 | 2012.35±1034.81 | 3438.78±2809.46 | 0.1116 |
| TNF-α | 737.11±178.53 | 801.83±295.88 | 0.5036 |

**Table S4 Group comparison of plasma levels of inflammatory cytokines between patients with/without apathy**

| Cytokine | Patients with apathy  (n=33) | Patients without apathy (n=6) | p-value |
| --- | --- | --- | --- |
| BAFF/TNFSF13B | 19431.14±6695.72 | 18871.83±5312.02 | 0.8479 |
| TWEAK/TNFSF12 | 1067.65±220.63 | 1020.37±212.39 | 0.6304 |
| sCD30/TNFRSF8 | 1911.50±485.29 | 1742.27±408.47 | 0.4279 |
| IFN-γ | 769.29±549.67 | 1082.70±1395.01 | 0.3358 |
| IL-10 | 530.54±315.38 | 534.96±255.20 | 0.9744 |
| IL-12p70 | 299.57±137.51 | 288.11±103.87 | 0.8477 |
| IL-17A | 953.72±1574.84 | 682.61±276.79 | 0.6797 |
| IL-1β | 581.76±431.40 | 5385.99±11872.90 | 0.4347 |
| IL-2 | 193.86±360.64 | 110.34±20.52 | 0.5782 |
| IL-4 | 31.49±17.63 | 38.93±12.79 | 0.3319 |
| IL-6 | 3148.11±2698.12 | 2422.34±922.97 | 0.5224 |
| TNF-α | 788.01±282.92 | 759.23±173.16 | 0.8120 |

**Table S5 Group comparison of plasma levels of inflammatory cytokines between patients with/without disinhibition**

| Cytokine | Patients with disinhibition  (n=16) | Patients without disinhibition  (n=23) | p-value |
| --- | --- | --- | --- |
| BAFF/TNFSF13B | 20035.20±9185.61 | 18865.02±3685.16 | 0.5837 |
| TWEAK/TNFSF12 | 1046.20±241.58 | 1070.23±203.80 | 0.7390 |
| sCD30/TNFRSF8 | 1825.94±444.55 | 1926.88±497.97 | 0.5197 |
| IFN-γ | 970.22±916.88 | 711.27±549.98 | 0.2774 |
| IL-10 | 499.25±259.54 | 553.45±335.10 | 0.5905 |
| IL-12p70 | **359.33±150.19** | **255.01±99.69** | **0.0129*** |
| IL-17A | 741.78±409.25 | 1030.43±1868.66 | 0.5485 |
| IL-1β | 3775.78±2987.84 | 1819.68±6076.51 | 0.4310 |
| IL-2 | 120.83±40.81 | 222.88±430.44 | 0.3525 |
| IL-4 | 34.34±17.00 | 31.45±17.36 | 0.6084 |
| IL-6 | **3775.78±****2987.84** | **1973.68±925.48** | **0.0255*** |
| TNF-α | 780.61±205.25 | 785.64±307.73 | 0.9548 |

**Table S6 Group comparison of plasma levels of inflammatory cytokines in different disease severity.**

| Cytokine | CDR® global =1  (n=18) | CDR® global =2  (n=15) | CDR® global =3  (n=6) | p-value |
| --- | --- | --- | --- | --- |
| BAFF/TNFSF13B | 17532.40±3917.33 | 2126.24±9098.33 | 19995.34±3113.37 | 0.2507 |
| TWEAK/TNFSF12 | 1057.82±275.18 | 1065.06±141.59 | 1056.32±212.64 | 0.9455 |
| sCD30/TNFRSF8 | 1829.38±399.63 | 1930.17±470.57 | 1941.95±720.43 | 0.7986 |
| IFN-γ | 778.55±870.72 | 882.30±637.97 | 772.39±491.52 | 0.9112 |
| IL-10 | 460.38±252.25 | 570.65±294.71 | 645.12±451.54 | 0.3642 |
| IL-12p70 | 282.84±133.18 | 314.83±135.52 | 300.11±136.12 | 0.7930 |
| IL-17A | **532.27±303.87** | **821.53±441.92** | **2277.41±3518.86** | **0.0324*** |
| IL-1β | 2130.01±6866.26 | 642.84±511.12 | 588.51±431.86 | 0.6172 |
| IL-2 | **124.38±40.37** | **121.60±44.52** | **499.43±827.64** | **0.0340*** |
| IL-4 | 30.42±17.14 | 35.59±16.93 | 31.89±21.10 | 0.6928 |
| IL-6 | 2569.80±1922.23 | 3101.27±2603.26 | 4274.39±3715.35 | 0.3617 |
| TNF-α | 733.04±217.08 | 776.53±199.06 | 952.82±477.73 | 0.2208 |

**Table S7 Group comparison of plasma levels of inflammatory cytokines in different severity of behavioral deficit.**

| Cytokine | FTLD-CDR behavior=1  (n=17) | FTLD-CDR-behavior=2  (n=15) | FTLD-CDR behavior=3  (n=7) | p-value |
| --- | --- | --- | --- | --- |
| BAFF/TNFSF13B | 17930.95±3642.32 | 20785.31±9437.02 | 19693.26±2952.35 | 0.4635 |
| TWEAK/TNFSF12 | 1066.30±281.22 | 1057.70±146.65 | 1051.72±194.49 | 0.9878 |
| sCD30/TNFRSF8 | 1811.51±404.44 | 1951.57±472.13 | 1923.43±659.48 | 0.6977 |
| IFN-γ | 797.93±893.51 | 863.33±646.87 | 766.85±448.94 | 0.9595 |
| IL-10 | 464.64±259.35 | 565.66±297.28 | 619.10±417.91 | 0.4623 |
| IL-12p70 | 286.20±136.50 | 303.93±135.67 | 312.87±128.77 | 0.8855 |
| IL-17A | **516.94±305.97** | **766.01±381.92** | **2184.30±3221.69** | **0.0290*** |
| IL-1β | 2240.42±7061.09 | 614.51±520.71 | 601.30±395.68 | 0.5697 |
| IL-2 | 124.43±41.61 | 120.06±43.99 | 449.04±767.21 | 0.0584 |
| IL-4 | 30.64±17.64 | 35.08±16.20 | 32.25±19.29 | 0.7708 |
| IL-6 | 2184.13±1039.81 | 3569.13±3010.80 | 3964.95±3489.05 | 0.1679 |
| TNF-α | 704.90±186.89 | 781.09±208.61 | 979.97±441.98 | 0.0687 |

**Table S8 Group comparison of plasma levels of inflammatory cytokines between patients with/without language deficit**

| Cytokine | With language deficit  (n=14) | Without language deficit  (n=25) | p-value |
| --- | --- | --- | --- |
| BAFF/TNFSF13B | 18078.27±3701.65 | 20054.5172±7539.1843 | 0.3651 |
| TWEAK/TNFSF12 | 1005.67±247.48 | 1091.0052±197.1725 | 0.2446 |
| sCD30/TNFRSF8 | 1882.28±349.84 | 1887.2540±537.1500 | 0.9754 |
| IFN-γ | 650.65±420.38 | 910.9444±841.4229 | 0.2871 |
| IL-10 | 508.12±257.39 | 544.1506±331.3912 | 0.7275 |
| IL-12p70 | 266.33±142.59 | 315.4269±124.7967 | 0.2700 |
| IL-17A | 555.53±334.40 | 1111.6310±1778.2342 | 0.2566 |
| IL-1β | 2512.33±7803.20 | 653.6498±473.8691 | 0.2379 |
| IL-2 | 132.51±47.27 | 208.17±414.28 | 0.5027 |
| IL-4 | 31.02±14.37 | 33.54±18.60 | 0.6649 |
| IL-6 | 2661.46±2225.96 | 3246.45±2680.21 | 0.4928 |
| TNF-α | 726.74±233.45 | 815.41±284.05 | 0.3269 |

**Table S9 Multiple regression analysis for associations between grey matter volume of left hippocampus and plasma levels of cytokines in FTD group.**

|  | SE | β | 95% CI | | P value |
| --- | --- | --- | --- | --- | --- |
|  |  |  | lower | upper |  |
| (Intercept) | 0.6388 |  | 1.9351 | 4.6308 | < .001 |
| BAFF/TNFSF13B | 1.3664×10^-5^ | 0.0588 | -2.3776×10^-5^ | 3.3880×10^-5^ | 0.7161 |
| TWEAK/TNFSF12 | 0.0005 | -0.1869 | -0.0017 | 0.0006 | 0.3153 |
| sCD30/TNFRSF8 | 0.0002 | 0.0660 | -0.0004 | 0.0006 | 0.7392 |
| IFN-γ | 0.0002 | -0.1456 | -0.0004 | 0.0002 | 0.4726 |
| IL-10 | 0.0004 | 0.3415 | -0.0002 | 0.0016 | 0.1134 |
| IL-12p70 | 0.0009 | 0.0039 | -0.0019 | 0.0019 | 0.9845 |
| IL-17A | **0.0002** | **-1.1783** | **-0.0009** | **-4.5272×10^-5^** | **0.0315*** |
| IL-1β | 0.0002 | -0.1963 | -0.0008 | 0.0002 | 0.2425 |
| IL-2 | 0.0008 | 0.7014 | -0.0006 | 0.0029 | 0.1798 |
| IL-4 | 0.0053 | 0.1999 | -0.0044 | 0.0180 | 0.2169 |
| IL-6 | 3.9620×10^-5^ | 0.1083 | -5.9289×10^-5^ | 0.0001 | 0.5477 |
| TNF-α | **0.0006** | **-0.6094** | **-0.0026** | **-7.4576×10^-5^** | **0.0391*** |

**Table S10 Multiple regression analysis for associations between grey matter volume of left para-hippocampus and plasma levels of cytokines in FTD group**

|  | SE | β | 95% CI | | P value |
| --- | --- | --- | --- | --- | --- |
|  |  |  | lower | upper |  |
| (Intercept) | 0.6894 |  | 1.8664 | 4.7756 | < 0.001 |
| BAFF/TNFSF13B | 1.4745×10^-5^ | 0.2167 | -1.2069×10^-5^ | 5.0151×10^-5^ | 0.2139 |
| TWEAK/TNFSF12 | 0.0006 | -0.2597 | -0.0020 | 0.0004 | 0.1908 |
| sCD30/TNFRSF8 | 0.0003 | 0.1502 | -0.0004 | 0.0008 | 0.4755 |
| IFN-γ | 0.0002 | -0.3286 | -0.0006 | 9.0815×10^-5^ | 0.1345 |
| IL-10 | 0.0004 | 0.4422 | -2.7049×10^-5^ | 0.0018 | 0.0562 |
| IL-12p70 | 0.0010 | 0.0236 | -0.0020 | 0.0022 | 0.9108 |
| IL-17A | 0.0002 | -1.0465 | -0.0009 | 2.8547×10^-5^ | 0.0650 |
| IL-1β | 0.0003 | -0.1991 | -0.0008 | 0.0002 | 0.2605 |
| IL-2 | 0.0009 | 0.6658 | -0.0008 | 0.0030 | 0.2251 |
| IL-4 | 0.0057 | 0.1235 | -0.0078 | 0.0164 | 0.4627 |
| IL-6 | 4.2757×10^-5^ | 0.2350 | -3.6255×10^-5^ | 0.0001 | 0.2240 |
| TNF-α | **0.0006** | **-0.6700** | **-0.0028** | **-0.0001** | **0.0324*** |

**Table S11 Multiple regression analysis for associations between grey matter volume of left amygdala and plasma levels of cytokines in FTD group.**

|  | SE | β | 95% CI | | P value |
| --- | --- | --- | --- | --- | --- |
|  |  |  | lower | upper |  |
| (Intercept) | 0.1844 |  | 0.3257 | 1.1039 | 0.0012 |
| BAFF/TNFSF13B | 3.9446×10^-6^ | 0.2463 | -2.6408×10^-6^ | 1.4004×10^-5^ | 0.1679 |
| TWEAK/TNFSF12 | 0.0002 | -0.1653 | -0.0005 | 0.0002 | 0.4065 |
| sCD30/TNFRSF8 | 7.1629×10^-5^ | 0.1736 | -9.1841×10^-5^ | 0.0002 | 0.4193 |
| IFN-γ | 4.5135×10^-5^ | -0.4022 | -0.0002 | 1.0003×10^-5^ | 0.0762 |
| IL-10 | 0.0001 | 0.4627 | -6.9898×10^-7^ | 0.0005 | 0.0506 |
| IL-12p70 | 0.0003 | -0.0245 | -0.0006 | 0.0005 | 0.9090 |
| IL-17A | 5.5866×10^-5^ | -0.9864 | -0.0002 | 1.5909×10^-5^ | 0.0856 |
| IL-1β | 6.8242×10^-5^ | -0.0883 | -0.0002 | 0.0001 | 0.6189 |
| IL-2 | 0.0002 | 0.7001 | -0.0002 | 0.0008 | 0.2112 |
| IL-4 | 0.0015 | 0.1883 | -0.0015 | 0.0050 | 0.2766 |
| IL-6 | 1.1438×10^-5^ | 0.3592 | -2.4780×10^-6^ | 4.5787×10^-5^ | 0.0755 |
| TNF-α | **0.0002** | **-0.7060** | **-0.0008** | **-5.1235×10^-5^** | **0.0276*** |

**Table S12 Multiple regression analysis for associations between grey matter volume of left superior temporal pole and plasma levels of cytokines in FTD group.**

|  | SE | β | 95% CI | | P value |
| --- | --- | --- | --- | --- | --- |
|  |  |  | lower | upper |  |
| (Intercept) | 0.7139 |  | 1.2432 | 4.2554 | 0.0013 |
| BAFF/TNFSF13B | 1.5268×10^-5^ | 0.3066 | -4.7741×10^-6^ | 5.9651×10^-5^ | 0.0901 |
| TWEAK/TNFSF12 | 0.0006 | -0.1765 | -0.0018 | 0.0007 | 0.3753 |
| sCD30/TNFRSF8 | 0.0003 | 0.1437 | -0.0004 | 0.0008 | 0.5015 |
| IFN-γ | 0.0002 | -0.2559 | -0.0006 | 0.0002 | 0.2452 |
| IL-10 | **0.0005** | **0.4700** | **1.4659×10^-5^** | **0.0020** | **0.0470*** |
| IL-12p70 | 0.0010 | -0.2765 | -0.0035 | 0.0008 | 0.2074 |
| IL-17A | 0.0002 | -1.0997 | -0.0009 | 1.5289×10^-5^ | 0.0573 |
| IL-1β | 0.0003 | -0.2569 | -0.0009 | 0.0002 | 0.1580 |
| IL-2 | 0.0009 | 0.7743 | -0.0006 | 0.0033 | 0.1681 |
| IL-4 | 0.0059 | 0.1289 | -0.0080 | 0.0171 | 0.4513 |
| IL-6 | 4.4272×10^-5^ | 0.3965 | -7.0000×10^-7^ | 0.0002 | 0.0516 |
| TNF-α | 0.0007 | -0.4529 | -0.0024 | 0.0004 | 0.1398 |

**Table S13 Multiple regression analysis for associations between grey matter volume of left middle temporal gyrus and plasma levels of cytokines in FTD group.**

|  | SE | β | 95% CI | | P value |
| --- | --- | --- | --- | --- | --- |
|  |  |  | lower | upper |  |
| (Intercept) | 2.4224 |  | 6.6282 | 16.8498 | < 0.001 |
| BAFF/TNFSF13B | 5.1810×10^-5^ | 0.1807 | -5.2059×10^-5^ | 0.0002 | 0.2846 |
| TWEAK/TNFSF12 | 0.0020 | -0.0905 | -0.0052 | 0.0033 | 0.6324 |
| sCD30/TNFRSF8 | 0.0009 | 0.3628 | -0.0003 | 0.0037 | 0.0883 |
| IFN-γ | 0.0006 | 0.0619 | -0.0011 | 0.0014 | 0.7651 |
| IL-10 | **0.0016** | **0.4814** | **0.0003** | **0.0069** | **0.0352*** |
| IL-12p70 | 0.0035 | -0.1892 | -0.0106 | 0.0041 | 0.3627 |
| IL-17A | **0.0007** | **-1.2450** | **-0.0033** | **-0.0002** | **0.0277*** |
| IL-1β | **0.0009** | **-0.5151** | **-0.0047** | **-0.0009** | **0.0067*** |
| IL-2 | 0.0031 | 0.9579 | -0.0008 | 0.0124 | 0.0806 |
| IL-4 | 0.0202 | 0.1969 | -0.0178 | 0.0673 | 0.2361 |
| IL-6 | **0.0002** | **-0.3866** | **-2.9629×10^-6^** | **-0.0006** | **0.0481*** |
| TNF-α | **0.0022** | **-0.6210** | **-0.0097** | **-0.0002** | **0.0407*** |

**Table S14 Multiple regression analysis for associations between grey matter volume of left inferior temporal gyrus and plasma levels of cytokines in FTD group.**

|  | SE | β | 95% CI | | P value |
| --- | --- | --- | --- | --- | --- |
|  |  |  | lower | upper |  |
| (Intercept) | 1.8777 |  | 5.6588 | 13.5822 | < 0.001 |
| BAFF/TNFSF13B | 4.0161×10^-5^ | 0.2398 | -2.2102×10^-5^ | 0.0001 | 0.1373 |
| TWEAK/TNFSF12 | 0.0016 | -0.2986 | -0.0060 | 0.0006 | 0.1056 |
| sCD30/TNFRSF8 | 0.0007 | 0.3307 | -0.0003 | 0.0028 | 0.0975 |
| IFN-γ | 0.0005 | -0.1005 | -0.0012 | 0.0007 | 0.6068 |
| IL-10 | 0.0012 | 0.2994 | -0.0007 | 0.0044 | 0.1484 |
| IL-12p70 | 0.0027 | -0.3164 | -0.0102 | 0.0012 | 0.1144 |
| IL-17A | **0.0006** | **-1.2855** | **-0.0027** | **-0.0003** | **0.0170*** |
| IL-1β | **0.0007** | **-0.4483** | **-0.0035** | **-0.0005** | **0.0109*** |
| IL-2 | 0.0024 | 0.9832 | -0.0002 | 0.0100 | 0.0585 |
| IL-4 | 0.0156 | 0.1781 | -0.0145 | 0.0514 | 0.2535 |
| IL-6 | **0.0001** | **-0.4131** | **-3.6197×10^-5^** | **-0.0005** | **0.0270*** |
| TNF-α | 0.0017 | -0.4760 | -0.0068 | 0.0005 | 0.0887 |

**Table S15 Multiple regression analysis for associations between grey matter metabolism of left olfactory gyrus and plasma levels of cytokines in FTD group.**

|  | SE | β | 95% CI | | P value |
| --- | --- | --- | --- | --- | --- |
|  |  |  | lower | upper |  |
| (Intercept) | 0.1305 |  | 0.6561 | 0.7579 | <0.001 |
| BAFF/TNFSF13B | 2.8100×10^-6^ | -0.1346 | 1.0046 | 1.5529 | 0.3649 |
| TWEAK/TNFSF12 | **0.0001** | **-0.3964** | **-8.5157×10^-6^** | **3.2915×10^-6^** | **0.0254*** |
| sCD30/TNFRSF8 | 5.0358×10^-5^ | -0.0260 | -0.0005 | -3.6460×10^-5^ | 0.8837 |
| IFN-γ | 3.1854×10^-5^ | 0.0078 | -0.0001 | 9.8324×10^-5^ | 0.9657 |
| IL-10 | 8.4583×10^-5^ | 0.2641 | -6.5531×10^-5^ | 6.8313×10^-5^ | 0.1722 |
| IL-12p70 | 0.0002 | -0.2392 | -5.7444×10^-5^ | 0.0003 | 0.2009 |
| IL-17A | **3.7843×10^-5^** | **-0.9806** | **-0.0006** | **0.0001** | **0.0372*** |
| IL-1β | 4.8064×10^-5^ | 0.0344 | -0.0002 | -5.6429×10^-6^ | 0.8165 |
| IL-2 | 0.0002 | 0.8091 | -8.9663×10^-5^ | 0.0001 | 0.0729 |
| IL-4 | 0.0011 | 0.0841 | -3.0969×10^-5^ | 0.0006 | 0.5582 |
| IL-6 | 7.8055×10^-6^ | 0.1549 | -0.0016 | 0.0029 | 0.3283 |
| TNF-α | **0.0001** | **-0.6376** | **-8.5564×10^-6^** | **2.4241×10^-5^** | **0.0166*** |

**Table S16** **Multiple regression analysis for associations between grey matter metabolism of left para-hippocampus and plasma levels of cytokines in FTD group.**

|  | SE | β | 95% CI | | P value |
| --- | --- | --- | --- | --- | --- |
|  |  |  | lower | upper |  |
| (Intercept) | 0.0885 |  | 0.9102 | 1.2823 | < 0.001 |
| BAFF/TNFSF13B | 1.9068×10^-6^ | 0.0790 | -2.8092×10^-6^ | 5.2030×10^-6^ | 0.5381 |
| TWEAK/TNFSF12 | **7.3420×10^-5^** | **-0.4195** | **-0.0004** | **-6.3711×10^-5^** | **0.0082*** |
| sCD30/TNFRSF8 | 3.4173×10^-5^ | -0.1200 | -9.8730×10^-5^ | 4.4858×10^-5^ | 0.4408 |
| IFN-γ | **2.1616×10^-5^** | **-0.3740** | **-9.7479×10^-5^** | **-6.6538×10^-6^** | **0.0269*** |
| IL-10 | 5.7397×10^-5^ | 0.1658 | -6.1657×10^-5^ | 0.0002 | 0.3182 |
| IL-12p70 | 0.0001 | -0.1028 | -0.0004 | 0.0002 | 0.5200 |
| IL-17A | 2.5680×10^-5^ | -0.6767 | -9.9835×10^-5^ | 8.0673×10^-6^ | 0.0908 |
| IL-1β | 3.2615×10^-5^ | 0.1045 | -4.1665×10^-5^ | 9.5380×10^-5^ | 0.4210 |
| IL-2 | 0.0001 | 0.2813 | -0.0001 | 0.0003 | 0.4558 |
| IL-4 | 0.0007 | 0.0236 | -0.0014 | 0.0017 | 0.8493 |
| IL-6 | 5.2967×10^-6^ | 0.1007 | -7.1459×10^-6^ | 1.5110×10^-5^ | 0.4619 |
| TNF-α | 7.8684×10^-5^ | -0.4083 | -0.0003 | 1.2239×10^-5^ | 0.0675 |

**Table S17 Multiple regression analysis for associations between grey matter metabolism of left amygdala and plasma levels of cytokines in FTD group.**

|  | SE | β | 95% CI | | P value |
| --- | --- | --- | --- | --- | --- |
|  |  |  | lower | upper |  |
| (Intercept) | 0.1327 |  | 0.9023 | 1.4599 | < 0.001 |
| BAFF/TNFSF13B | 2.8579×10^-6^ | -0.0727 | -7.3784×10^-6^ | 4.6299×10^-6^ | 0.6364 |
| TWEAK/TNFSF12 | 0.0001 | -0.1108 | -0.0003 | 0.0002 | 0.5219 |
| sCD30/TNFRSF8 | **5.1216×10^-5^** | **-0.4352** | **-0.0002** | **-1.4315×10^-5^** | **0.0285*** |
| IFN-γ | 3.2396×10^-5^ | -0.0276 | -7.2849×10^-5^ | 6.3275×10^-5^ | 0.8842 |
| IL-10 | 8.6023×10^-5^ | 0.2069 | -8.8929×10^-5^ | 0.0003 | 0.3000 |
| IL-12p70 | 0.0002 | -0.0265 | -0.0004 | 0.0004 | 0.8897 |
| IL-17A | 3.8487×10^-5^ | -0.1899 | -9.6925×10^-5^ | 6.4792×10^-5^ | 0.6813 |
| IL-1β | 4.8882×10^-5^ | 0.1751 | -4.6551×10^-5^ | 0.0002 | 0.2658 |
| IL-2 | 0.0002 | -0.2611 | -0.0004 | 0.0002 | 0.5631 |
| IL-4 | 0.0011 | 0.0352 | -0.0021 | 0.0026 | 0.8138 |
| IL-6 | 7.9384×10^-6^ | -0.0110 | -1.7218×10^-5^ | 1.6137×10^-5^ | 0.9465 |
| TNF-α | 0.0001 | -0.3176 | -0.0004 | 9.9192×10^-5^ | 0.2238 |

**Table S18 Multiple regression analysis for associations between grey matter metabolism of left middle temporal pole and plasma levels of cytokines in FTD group.**

|  | SE | β | 95% CI | | P value |
| --- | --- | --- | --- | --- | --- |
|  |  |  | lower | upper |  |
| (Intercept) | 0.1611 |  | 0.5769 | 1.2537 | < .001 |
| BAFF/TNFSF13B | 3.4687×10^-6^ | 0.3361 | -6.6510×10^-8^ | 1.4509×10^-5^ | 0.0519 |
| TWEAK/TNFSF12 | **0.0001** | **-0.3814** | **-0.0006** | **-4.1446×10^-7^** | **0.0497*** |
| sCD30/TNFRSF8 | 6.2163×10^-5^ | 0.0203 | -0.0001 | 0.0001 | 0.9186 |
| IFN-γ | 3.9321×10^-5^ | -0.2791 | -0.0001 | 2.7513×10^-5^ | 0.1782 |
| IL-10 | 0.0001 | 0.1432 | -0.0001 | 0.0003 | 0.4981 |
| IL-12p70 | **0.0002** | **-0.4239** | **-0.0010** | **-2.0931×10^-6^** | **0.0491*** |
| IL-17A | 4.6714×10^-5^ | -0.5838 | -0.0002 | 4.2022×10^-5^ | 0.2452 |
| IL-1β | 5.9331×10^-5^ | -0.1410 | -0.0002 | 7.3291×10^-5^ | 0.3981 |
| IL-2 | 0.0002 | 0.4484 | -0.0002 | 0.0006 | 0.3562 |
| IL-4 | 0.0013 | 0.0039 | -0.0028 | 0.0029 | 0.9803 |
| IL-6 | 9.6352×10^-6^ | 0.2101 | -8.4666×10^-6^ | 3.2019×10^-5^ | 0.2374 |
| TNF-α | 0.0001 | -0.2229 | -0.0004 | 0.0002 | 0.4188 |

**Table S19 Multiple regression analysis for associations between grey matter metabolism of left inferior temporal gyrus and plasma levels of cytokines in FTD group.**

|  | SE | β | 95% CI | | P value |
| --- | --- | --- | --- | --- | --- |
|  |  |  | lower | upper |  |
| (Intercept) | 0.1195 |  | 0.8453 | 1.3473 | <0.001 |
| BAFF/TNFSF13B | **2.5726×10^-6^** | **-0.3827** | **-6.3477×10^-7^** | **-1.1444×10^-5^** | **0.0305*** |
| TWEAK/TNFSF12 | **9.9054×10^-5^** | **-0.4181** | **-0.0004** | **-1.8144×10^-5^** | **0.0347*** |
| sCD30/TNFRSF8 | 4.6104×10^-5^ | 0.0827 | -7.7532×10^-5^ | 0.0001 | 0.6800 |
| IFN-γ | 2.9163×10^-5^ | -0.0183 | -6.3928×10^-5^ | 5.8608×10^-5^ | 0.9283 |
| IL-10 | 7.7437×10^-5^ | -0.1915 | -0.0002 | 9.1781×10^-5^ | 0.3719 |
| IL-12p70 | 0.0002 | -0.3993 | -0.0007 | 2.2769×10^-5^ | 0.0646 |
| IL-17A | 3.4646×10^-5^ | -0.6126 | -0.0001 | 2.9527×10^-5^ | 0.2278 |
| IL-1β | 4.4003×10^-5^ | -0.3167 | -0.0002 | 7.6697×10^-6^ | 0.0700 |
| IL-2 | 0.0001 | 0.3764 | -0.0002 | 0.0004 | 0.4413 |
| IL-4 | 0.0010 | -0.0041 | -0.0021 | 0.0021 | 0.9798 |
| IL-6 | 7.1461×10^-6^ | 0.0968 | -1.1029×10^-5^ | 1.8998×10^-5^ | 0.5840 |
| TNF-α | 0.0001 | 0.0409 | -0.0002 | 0.0002 | 0.8820 |

**Table S20 Multiple regression analysis for associations between CDR® plus NACC FTLD sum of box and plasma levels of cytokines in FTD group.**

|  | SE | β | 95% CI | | P value |
| --- | --- | --- | --- | --- | --- |
|  |  |  | lower | upper |  |
| (Intercept) | 0.9296 |  | 7.7185 | 11.4893 | <0.001 |
| IL-17A | **0.0014** | **0.9285** | **0.0004** | **0.0063** | **0.0284*** |
| IL-2 | 0.0063 | -0.6755 | -0.0233 | 0.0023 | 0.1054 |

**Table S21 Multiple regression analysis for associations between CDR® sum of box and plasma levels of cytokines in FTD group.**

|  | SE | β | 95% CI | | P value |
| --- | --- | --- | --- | --- | --- |
|  |  |  | lower | upper |  |
| (Intercept) | 0.3881 |  | 0.0888 | 1.6644 | 0.0302 |
| BAFF/TNFSF13B | 1.7170×10^-5^ | 0.2465 | -6.8838×10^-6^ | 6.2832×10^-5^ | 0.1122 |
| IL-17A | **8.0947×10^-5^** | **0.3472** | **1.0450×10^-5^** | **0.0003** | **0.0378** |
| IL-6 | 4.6875×10^-5^ | 0.1303 | -5.7239×10^-5^ | 0.0001 | 0.4240 |

**Table S22 Multiple regression analysis for associations between BNT and plasma levels of cytokines in FTD group.**

|  | SE | β | 95% CI | | P value |
| --- | --- | --- | --- | --- | --- |
|  |  |  | lower | upper |  |
| (Intercept) | 5.9805 |  | 14.6048 | 38.9685 | <0.001 |
| TWEAK/TNFSF12 | 0.0049 | -0.2095 | -0.0168 | 0.0033 | 0.1816 |
| IFN-γ | 0.0015 | -0.2537 | -0.0055 | 0.0006 | 0.1125 |
| IL-17A | **0.0020** | **-0.9305** | **-0.0086** | **-0.0004** | **0.0327** |
| IL-1β | **0.0002** | **-0.3351** | **-0.0010** | **-4.5202×10^-5^** | **0.0323** |
| IL-2 | 0.0086 | 0.6934 | -0.0028 | 0.0320 | 0.0980 |
| IL-4 | 0.0641 | 0.1674 | -0.0618 | 0.1991 | 0.2918 |

**Table S23 Multiple regression analysis for associations between FBI apathy scores and plasma levels of cytokines in FTD group.**

|  | SE | β | 95% CI | | P value |
| --- | --- | --- | --- | --- | --- |
|  |  |  | lower | upper |  |
| (Intercept) | 7.8598 |  | -17.9633 | 14.0184 | 0.8034 |
| BAFF/TNFSF13B | **0.0002** | **0.5269** | **0.0003** | **0.0010** | **< .001** |
| TWEAK/TNFSF12 | 0.0054 | 0.1614 | -0.0048 | 0.0172 | 0.2632 |
| IL-10 | 0.0038 | 0.1000 | -0.0050 | 0.0104 | 0.4765 |
| IL-12p70 | 0.0089 | -0.2481 | -0.0338 | 0.0026 | 0.0901 |
| IL-6 | 0.0005 | 0.1810 | -0.0003 | 0.0015 | 0.1990 |

**Table S24 Multiple regression analysis for associations between FBI total score**

**and plasma levels of cytokines in FTD group.**

|  | SE | β | 95% CI | | P value |
| --- | --- | --- | --- | --- | --- |
|  |  |  | lower | upper |  |
| (Intercept) | 9.5118 |  | -14.9733 | 23.6083 | 0.6526 |
| BAFF/TNFSF13B | **0.0003** | **0.3703** | **0.0001** | **0.0012** | **0.0211** |
| TWEAK/TNFSF12 | 0.0079 | 0.1199 | -0.0099 | 0.0222 | 0.4399 |

**Table S25 Multiple regression analysis for associations between disease duration and plasma levels of cytokines in FTD group.**

|  | SE | β | 95% CI | | P value |
| --- | --- | --- | --- | --- | --- |
|  |  |  | lower | upper |  |
| (Intercept) | 5.7723 |  | 27.7228 | 51.1363 | < 0.001 |
| IFN-γ | 0.0034 | -0.1450 | -0.0102 | 0.0037 | 0.3503 |
| IL-4 | **0.1460** | **-0.3646** | **-0.6434** | **-0.0512** | **0.0228*** |
